# A Hybrid Approach to Industrial Augmented Reality Using Deep Learning-Based Facility Segmentation and Depth Prediction

**DOI:** 10.3390/s21010307

**Published:** 2021-01-05

**Authors:** Minseok Kim, Sung Ho Choi, Kyeong-Beom Park, Jae Yeol Lee

**Affiliations:** 1 Korea Institute of Science and Technology Information (KISTI), Daejeon 34141, Korea; kimminseok@kisti.re.kr; 2 Department of Industrial Engineering, Chonnam National University, Gwangju 61186, Korea; 177541@jnu.ac.kr (S.H.C.); 188566@jnu.ac.kr (K.-B.P.)

**Keywords:** augmented reality (AR), deep learning, facility segmentation, industrial AR, manufacturing information visualization

## Abstract

Typical AR methods have generic problems such as visual mismatching, incorrect occlusions, and limited augmentation due to the inability to estimate depth from AR images and attaching the AR markers onto physical objects, which prevents the industrial worker from conducting manufacturing tasks effectively. This paper proposes a hybrid approach to industrial AR for complementing existing AR methods using deep learning-based facility segmentation and depth prediction without AR markers and a depth camera. First, the outlines of physical objects are extracted by applying a deep learning-based instance segmentation method to the RGB image acquired from the AR camera. Simultaneously, a depth prediction method is applied to the AR image to estimate the depth map as a 3D point cloud for the detected object. Based on the segmented 3D point cloud data, 3D spatial relationships among the physical objects are calculated, which can assist in solving the visual mismatch and occlusion problems properly. In addition, it can deal with a dynamically operating or a moving facility, such as a robot—the conventional AR cannot do so. For these reasons, the proposed approach can be utilized as a hybrid or complementing function to existing AR methods, since it can be activated whenever the industrial worker requires handing of visual mismatches or occlusions. Quantitative and qualitative analyses verify the advantage of the proposed approach compared with existing AR methods. Some case studies also prove that the proposed method can be applied not only to manufacturing but also to other fields. These studies confirm the scalability, effectiveness, and originality of this proposed approach.

## 1. Introduction

Recently, many studies have been attempted to increase productivity by building smart factories through the convergence of the Internet of Things (IoT) and information and communication technology (ICT) in traditional manufacturing systems [1,2]. As the IoT technology is used to connect machines, sensors, and facilities seamlessly, smartization can be realized, and the role of the worker has been changed from an operator to a smart manager [3,4]. Therefore, it is essential to provide the right manufacturing information to the smart manager concerning his or her role for practical task assistance.

Augmented reality is considered to provide user-centric information more easily in various environments by embedding visual information onto the real objects directly. In particular, the AR-based visualization of manufacturing information, called industrial AR, can provide more effective task assistance, which can reduce a worker’s cognitive load and increase work efficiency [5,6,7].

**Figure 1:**
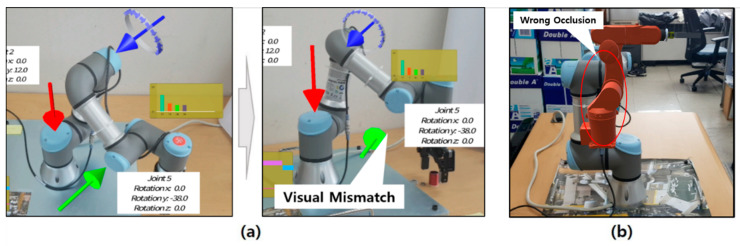
Visualization problems in conventional industrial AR systems: (**a**) visual mismatch occurs when the physical configuration of the robot is changed; (**b**) undesired occlusion occurs when the depth perception of physical and virtual objects cannot be properly estimated (the virtual object is located behind the real robot.).

Typical industrial AR superimposes necessary manufacturing data around real equipment using AR markers for indirect object detection and visual registration. However, it is difficult to attach AR markers on the physical objects such as machines and facilities whose shapes are complicated. It is much more difficult to attach them if the configuration of the facility or equipment is dynamically moving during the operation. Note that it is challenging to acquire a 3D spatial relationship between physical objects from an RGB image using only one AR marker. Thus, it is necessary to attach multiple AR markers to them to solve this problem. However, it is sometimes infeasible to attach multiple markers. In addition, some of the markers cannot be adequately detected due to spatial occlusion when the viewpoint is changed. Therefore, visual mismatch and incorrect occlusion problems still occur in industrial AR environments, as shown in Figure 1. These visual problems increase cognitive load and prevent the worker from understanding the required task quickly and accurately at the right time.

For example, although the manufacturing information can be visualized correctly from a particular viewpoint of the worker, as shown in Figure 1a, the visual mismatch occurs when the viewpoint is changed. Besides, when the physical configuration of a facility such as a robot is changed, incorrect information is displayed to the worker. Furthermore, as shown in Figure 1b, the equipment located near the worker is occluded by the AR view related to the far-off object irrespective of the distance between the worker and the actual equipment. Both examples indicate the inherent limitation of currently available AR systems.

For this reason, several previous studies have been conducted to support effective and user-friendly visualization by solving these problems in industrial AR [8,9,10,11]. However, most of them could not conduct object detection and find 3D spatial relations among different physical objects effectively in industrial AR. In particular, they could not correctly recognize the configuration of a dynamically moving object even if the AR marker was attached or area learning-based AR was used [10]. One way to figure out the 3D spatial relations among facilities is to use a depth camera to scan them and acquire a 3D point cloud. However, most of the smart devices, such as smartphones and tablets, do not have depth cameras. While a depth camera is attached, AR markers are still required to recognize real objects [12].

**Figure 2:**
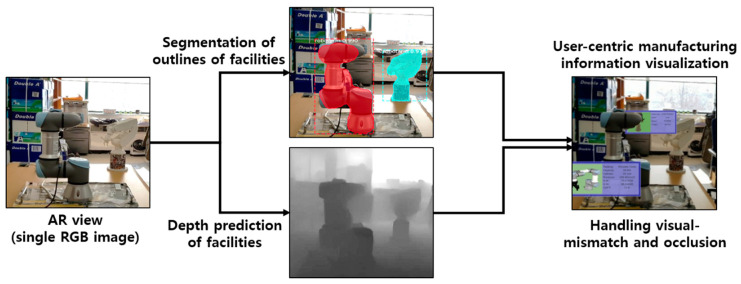
Proposed industrial AR using facility segmentation and depth prediction.

In this study, we propose a hybrid approach to industrial AR, which can complement existing AR methods and provide manufacturing information more effectively through the deep learning-based instance segmentation and depth prediction of physical objects in the AR scene. Deep learning-based instance segmentation is performed to recognize physical facilities and to outline their regions in the AR view (e.g., 2D RGB image), and the depth prediction using another deep learning is simultaneously conducted to predict a 3D depth map of those facilities from the same AR view, as shown in Figure 2. Then, by combining the segmented regions of the real facilities and the 3D depth map, the 3D point clouds of the facilities and their 3D spatial relations are also segmented and constructed. Thus, the proposed industrial AR approach can not only solve the visual mismatch problem between manufacturing information and actual facilities, but also can handle occlusion properly, which is used to provide more user-friendly manufacturing visualization to improve the worker’s understanding of the environment. In addition to stationary objects, dynamically moving objects such as robots can also be handled to provide occlusion-free manufacturing visualization through facility segmentation and depth prediction.

The contributions of this study are as follows.

(1) A new industrial AR method is proposed to provide manufacturing information more intuitively and accurately by solving visual mismatch and occlusion problems through the deep learning-based instance segmentation and depth prediction.
(2) It is possible to handle dynamically moving facilities and visualize the manufacturing information more effectively.
(3) The proposed approach can complement existing AR methods. Thus, it can be utilized as a hybrid or complementing function to existing AR methods, since it can be activated whenever the industrial worker requires handing of visual mismatches or occlusions.
(4) The applicability and extensibility of this study can be confirmed through experiments and various viable implementations.

The paper is organized as follows. Section 2 describes related research. Section 3 presents the proposed industrial AR method. Section 4 describes several implementation results of the proposed approach. Quantitative and qualitative analyses are conducted and evaluated in Section 5. Section 6 discusses the proposed approach. Finally, Section 7 concludes the paper and presents future studies.

## 2. Related Work

### 2.1. Manufacturing Information Visualization and Task Assistance in Industrial AR

Previous studies have shown that AR-based task assistance methods can reduce the worker’s mistakes more than text or video-based methods. Additionally, it is well-known that the provision of manufacturing information using AR is more effective in supporting the task of the worker [13,14,15].

Several previous AR studies have been conducted to provide useful and effective visualizations of manufacturing information, such as the real-time status of the facility or guided instructions with single or multiple AR markers [16,17,18,19]. Maris et al. [19] implemented an AR-based system to support collaborative assembly between the worker and the robot. Through this implementation, different manufacturing information was provided through functions such as assistance for the assembly process and the working space and trajectory visualization of the robot. Gattullo et al. [20] proposed a method of transforming a manual created with existing visualization methods (text, PDF file, etc.) into the AR-based visualization. Michalos et al. [21] also developed an AR-based system that supported collaborative work in environments where workers and robots were located together. The system allowed the visualization of the assembly process, video and text-based manuals, and status information. Tzimas et al. [22] proposed an AR method to support workers to set attribute values for various machines and parts in a manufacturing system.

To visualize manufacturing information in AR environments, methods using markerless tracking systems without AR markers were also studied. Lima et al. [23] proposed a tracking system using image feature points captured by smart devices rather than feature points of the markers to support AR-based applications for the maintenance and repair of automobiles. The proposed approach encompasses scene modeling, system calibration, and tracking steps for markerless tracking using an RGB-D sensor. Wang et al. [24] proposed an AR-based assembly assistance method using a coarse-to-fine markerless tracking system. The proposed approach was divided into two stages: the offline preparation stage and the online execution stage.

### 2.2. Dynamic Visualization by Handling Occlusion between Real Objects and Virtual Objects in AR

In typical AR, a real object and its corresponding virtual model are simultaneously visualized on the screen of a smart or wearable device. Due to the inherent rendering characteristic of AR, real objects on the AR view are obscured by virtual objects. In particular, the incorrect occlusion lowers the worker’s immersive feeling and also hinders task assistance in various industrial AR environments. Therefore, several methods have been proposed for handling occlusions between real and virtual objects in AR properly. Those can be divided into three approaches, a contour-based approach, a depth-based approach, and a 3D reconstruction-based approach.

The contour-based approach traces the silhouette or edge of the object to handle the occlusion [8]. The contour of a real object is extracted by user interaction or extracted automatically by analyzing the attribute of the object. The contour-based approach cannot effectively deal with 3D spatial relations. The depth-based approach uses an RGB-D camera to acquire depth information of the real object and environment. By comparing the depth value of each pixel corresponding to a real object and the depth value of a pixel corresponding to a virtual object in an image, it is possible to determine the 3D spatial relation between them. Based on the spatial relation, it is possible to handle occlusion problems [9,10,25]. However, the quality of the depth information depends on the material of the object. In addition, most of the currently available AR devices do not support RGB-D cameras, which limits the applicability of the industrial AR in real manufacturing environments. The 3D reconstruction-based approach creates a 3D model of a real object in advance. After that, the 3D model of the real object is compared with the depth value of the virtual object to handle occlusion [11,26,27]. This approach can construct a 3D environment using an RGB-D camera or geographic information system (GIS) database.

In particular, most of the previous studies were mainly applicable to a static environment wherein the real object could not move. In addition, most mobile devices do not have RGB-D sensors. Therefore, this study proposes a new industrial AR method using a single RGB image to handle visual mismatches and occlusions using deep learning in dynamic environments [28].

### 2.3. Industrial IoT and AR Applications

IoT integrates the key technologies of industrial communication, computing, and control, and is providing a new way for a wide range of manufacturing resources to optimize management and dynamic scheduling [29]. Recently, IoT and cloud computing have been widely applied in various industrial fields, as they can provide new methods for intelligent perception and connection from M2M (including man-to-man, man-to-machine, and machine-to-machine), and on-demand use and efficient sharing of resources, respectively [30]. Thus, IoT is considered as one of the most important key technologies in intelligent manufacturing in the context of Industry 4.0 [31].

AR also plays an important role in IoT applications, such as smart homes, farming, and robot task planning. Seo et al. proposed a hybrid reality-based user experience and evaluation of a context-aware smart home [32]. The user experience is provided by the integration of egocentric virtual reality and exocentric augmented reality. Huo et al. presented Scenariot, a method that enables instant discovery and localization of the surrounding smart things while also spatially registering them with a mobile AR system [33]. An AR-IoT system was proposed to superimpose IoT data directly on real-world objects and to enhance object interaction for crop monitoring [34]. Cao et al. presented V.Ra, a visual and spatial programming system for robot-IoT task authoring [35]. Jo et al. proposed a scalable AR framework enabled by simple extension to the IoT infrastructure for interacting with IoT appliances everywhere [36]. Although most previous studies have shown promising directions of utilizing AR in IoT environments, they have not dealt with handling incorrect occlusion and visual mismatch for providing more intuitive and easy-understandable AR information for industrial workers. The primary objective of this research is to solve these problems in industrial AR.

## 3. Industrial AR Using Deep Learning-Based Facility Segmentation and Depth Prediction

### 3.1. Overview of the Proposed Approach

**Figure 3:**
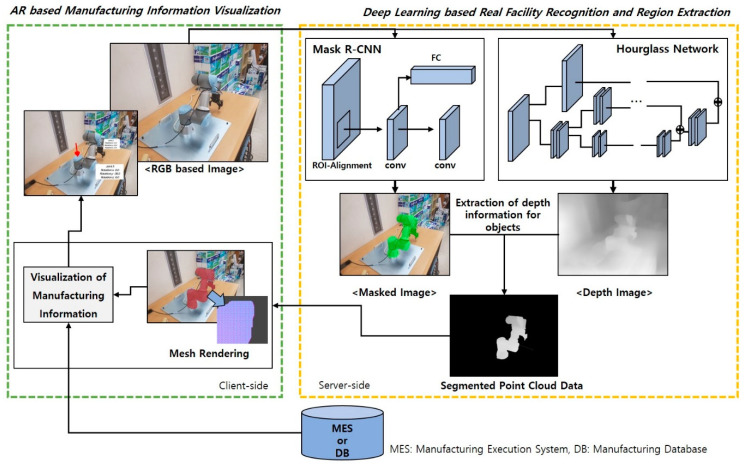
Framework of the proposed industrial AR.

This study proposes a new hybrid approach to industrial AR that can provide user-friendly manufacturing information to the worker more effectively and naturally by combining deep learning and augmented reality, which can complement existing AR methods. Instance segmentation is applied to the image of the AR view to detect actual equipment and tools and to segment their outlines from the AR image. Besides, depth information is predicted at the same time, using another deep learning approach. Then, the relative 3D spatial relationship between objects is calculated using the segmented object instances and predicted depth information. Finally, the manufacturing information is augmented on physical objects so that the worker can easily understand their tasks through AR scenes. Figure 3 shows a schematic diagram of the proposed industrial AR, which consists of (1) deep learning-based facility recognition and region extraction and (2) AR-based manufacturing information visualization modules.

The deep learning-based facility recognition and region extraction module applies the mask region-based convolutional neural network (Mask R-CNN [37]) to the RGB image captured by the AR camera, which detects actual facilities or tools and then segments the surrounding regions of the detected objects from the RGB image. At the same time, depth prediction is conducted to estimate the 3D depth map of the same AR image [38,39]. Then, the results of instance segmentation and depth prediction are combined to obtain a 3D point cloud of the detected facilities and spatial relationship among them.

The AR-based manufacturing information visualization module superimposes the required information onto the detected objects for task assistance. Firstly, it creates triangle meshes using the segmented and predicted 3D point cloud data. Then, a 3D spatial relation among real facilities is calculated from the constructed 3D meshes as the 3D meshes can be sorted in the ascending order in the depth map. Finally, a meshing rendering using a depth mask shader is applied to the 3D meshes for occlusion rendering, which makes the region of the object in the AR view depth-aware transparent. For example, the far object instance can be occluded by the near object instance, although there is no such information in the AR scene.

**Figure 4:**
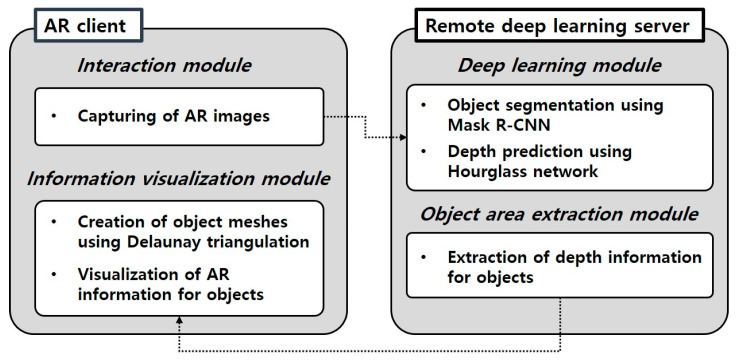
AR client and remote deep learning server.

The deep learning methods used in this study are difficult to be executed in mobile or wearable devices directly because of their hardware and software restrictions. For this reason, a client-server module was developed to solve this problem. The module consists of the AR client and the remote deep learning server, as shown in Figure 4. Smart devices or wearable devices play the role of the AR client. Firstly, the RGB-based AR image captured from the smart device is transmitted to the remote deep learning server through a network connection. The remote deep learning server is used to achieve instance segmentation and depth prediction of the physical facility in the AR image. Results are combined to generate the segmented 3D point cloud data of the physical object. Then, they are transmitted to the AR client. The AR client generates 3D meshes based on the segmented 3D point cloud. It also conducts the occlusion rendering to handle incorrect occlusions and solve visual mismatches. Finally, the AR client overlays manufacturing information on the AR display.

Although this study can solve the problems of existing AR, such as visual mismatches and incorrect occlusions, with deep learning it is difficult to guarantee real-time performance regarding providing manufacturing information to users. Therefore, the proposed approach can be utilized as a hybrid or complementing function to existing AR methods since it can be activated whenever the industrial worker requires handing of visual mismatches or occlusions. In other words, the proposed method and the existing AR method can be used at the same time, so that they can be switched with each other as needed, thereby complementing each other. Since the technology of deep learning and the performances of mobile devices are rapidly developing, it is expected that deep learning will be executed in real time on smart devices rather than via server–client connections sooner or later.

Note that the proposed approach can not only detect physical object instances but also segment the outlines of the objects whose configurations are dynamically moving and changing. Most of the previous AR works have not considered this issue. In addition, manufacturing information is augmented in the right position by overcoming visual mismatches and incorrect occlusions. For example, visual information of a certain facility that is far from the worker can be obscured by nearby facilities. Besides, manufacturing information of a nearby facility can be displayed in a larger and detailed form, and that of a remote facility can be displayed in a simplified and small form, which can provide a more intuitive and user-centric visual form.

### 3.2. Detection, Segmentation, and Depth Prediction of Real Facilities

It is crucial to acquire 3D spatial information and the class and position of each equipment to provide manufacturing information appropriate for task assistance in mobile AR.

#### 3.2.1. Object Detection and Facility Instance Segmentation

**Figure 5:**
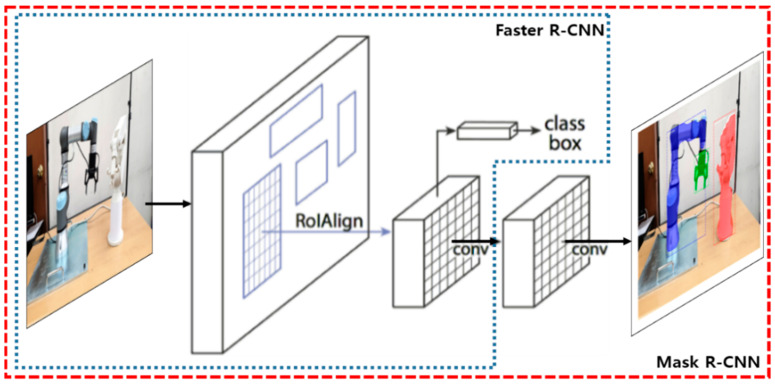
Mask R-CNN-based segmentation of manufacturing facilities.

Object instance segmentation identifies object outlines at the pixel level by detecting objects and segmenting their regions in the RGB image, as shown in Figure 5. Then, the object can be rendered in the pixel level according to the outline of the recognized object shape. For example, it can segment Universal Robot-3^TM^ (UR3) and its gripper from the AR view. Unlike object detection, which displays a detected object as a bounding box, instance segmentation accurately can render the region corresponding to the outline of the detected object. In particular, it recognizes the region as another object, even if it is classified into the same class. In this study, instance segmentation was conducted using the Mask R-CNN [37] to outline the regions for real facilities from the AR image acquired through smart devices, as shown in Figure 5.

Mask R-CNN extends Faster R-CNN [40] by adding a branch for predicting segmentation masks on each region of interest (RoI), in parallel with the existing branch for classification and bounding box regression [37]. The mask branch is a fully convolutional network (FCN) applied to each RoI, predicting a segmentation mask in a pixel-to-pixel manner. Mask R-CNN has two stages for instance segmentation. The first stage scans the image and generates region proposals. The second stage classifies the proposals and generates bounding boxes and masks. In the first stage, the region proposal network (RPN), like Faster R-CNN, is used to predict the bounding box for the object. However, in Mask R-CNN, RoIAlign was used instead of RoIPool for accurate segmentation and fixing misalignment.

Mask R-CNN defines a multi-task loss on each sampled RoI as follows (Equation (1)).

(1) $$L=\;L_{cls}+\;L_{box}+\;L_{mask}$$

The classification loss $L_{cls}$ and the bounding-box loss $L_{box}$ (Equation (2)) are identical as those defined in Faster R-CNN [40] as follows.

(2) $$L_{cls}+\;L_{box}=L\left(p,\;u,\;t^{u},\;v\right)\;=\;L_{cls}\left(p,\;u\right)+\;\lambda \left[u\;\geq 1\right]L_{loc}\left(t^{u},\;v\right)$$

where *p* and *t* represent the size and position of the predicted classes and bounding box, and *u* and *v* represent the size and position of the real class and bounding box.

Finally, the mask loss $L_{mask}$ is defined as the average binary cross-entropy.

#### 3.2.2. Depth Prediction

**Figure 6:**
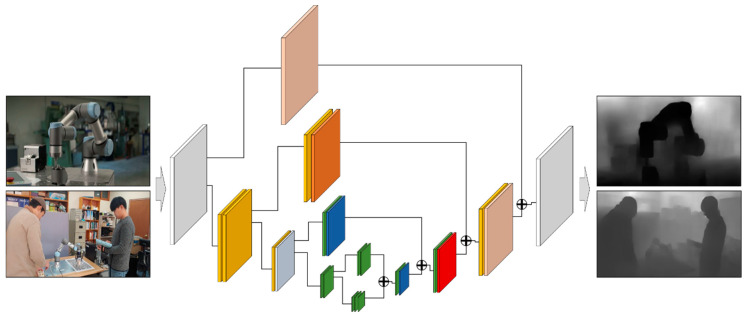
Depth prediction of object instances from the AR image for calculating 3D spatial relation.

By conducting the instance segmentation mentioned above, it is possible to calculate 2D spatial relations, such as relative positions between real objects. However, since the depth information is unknown, a 3D spatial relation cannot be derived, which can cause visual mismatch and occlusion problems. To calculate the 3D spatial relationships between objects from the AR view, depth information must be predictable. Note that mobile AR also utilizes a single image so that it cannot percept depth information from the single image without AR markers. In this study, depth information was predicted through an hourglass network by training MageDepth Dataset [38,39]. Depth prediction aims to recover depth information from a single RGB image taken from a camera. As shown in Figure 6, the hourglass network consists of a series of convolutions using a variant of the inception model and down-sampling, followed by a series of convolutions and up-sampling, interleaved with skip connections that add back features. The symmetric shape of the network resembles an hourglass. Through this process, the depth information of the RGB image can be acquired, and the resolution of the depth information is the same as that of the RGB image.

The scale-invariant loss function *L* (Equation (3)) combines three terms as follows [38].

(3) $$L=\;L_{data}+\;\alpha L_{grad}+\;\beta L_{ord}$$

The scale-invariant data loss $L_{data}$ (Equation (4)) calculates the mean square error of the difference between all pairs of log-depths. Let *L* be a predicted log-depth map and $L^{*}$ a ground truth long-depth map. $L_{i}$ and $L_{i}^{*}$ denote corresponding individual log-depth values indexed by pixel position *i*.

(4) Ldata= 1n∑(Ri)i=1n2− 1n2(∑Ri=1ni)2

where $R_{i}=\;L_{i}-\;L_{i}^{*}$ and *n* is the number of valid depths in the ground truth depth map.

The scale-invariable gradient matching loss $L_{grad}$ (Equation (5)) makes gradient changes smoother and depth discontinuities sharper in the predicted depth map. It is defined as an *L_1_* penalty on differences in log-depth gradients between the predicted and ground truth depth map as follows.

(5) $$L_{grad}=\;\frac{1}{n}\underset{k}{\sum }\underset{i}{\sum }\left(\left|\nabla_{x}R_{i}^{k}\right|+\left|\nabla_{y}R_{i}^{k}\right|\right)$$

where $R_{i}^{k}$ is the value of the log-depth difference map at position *i* and scale *k*.

The ordinal depth loss $L_{ord}$ (Equation (6)) encourages the predicted depth map to agree with the ground-truth ordinal relations as follows.

(6) $$L_{ord}=\;\{\begin{array}{c} \log\left(1+exp\left(P_{ij}\right)\right)\;\;\;\;\;\;if\;P_{ij}\;\leq \;\tau \\ \log\left(1+exp\left(\sqrt{P_{ij}}\right)\right)+c\;\;\;\;if\;P_{ij}\;>\;\tau \end{array}$$

where $P_{ij}=-r_{ij}^{*}\left(L_{i}-L_{j}\right)$ and $r_{ij}^{*}$ is the automatically labeled ordinal depth relation between *i* and *j* ($r_{ij}^{*}=1$ if pixel *i* is further than *j* and −1 otherwise).

As a result, the segmented 3D point cloud data of the detected facilities can be acquired by combining the predicted depth information with the outcome of instance segmentation. Since the dimension of the predicted information is the same as that of the RGB image, the segmentation of 3D point clouds corresponding to the detected facilities from the predicted depth map is straightforward.

### 3.3. Industrial AR-Based Manufacturing Information Visualization

**Figure 7:**
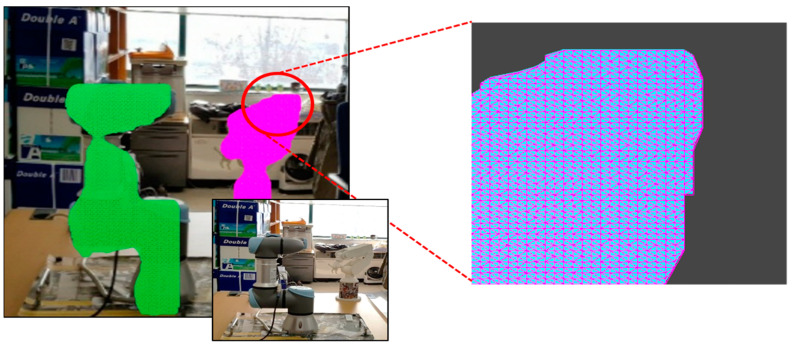
3D mesh construction for mesh rendering to deal with incorrect occlusions.

When information on the segmented 3D point cloud data is transmitted to the mobile device of the worker together with the classification information of the detected facilities, all the information is combined to handle visual mismatches and incorrect occlusions in manufacturing information visualization. Since segmented point cloud data do not contain surface information for rendering and occlusion handling, a triangular mesh is constructed from the segmented point cloud of each object instance. If there are multiple detected object instances, several triangular meshes are generated. Each triangular mesh can represent a detected object in the 3D AR space. A Delaunay triangulation was applied to construct a triangular mesh of the segmented 3D point cloud of each object instance [41,42], as shown in Figure 7. A meshing rendering using a depth mask shader is applied to the triangular mesh for occlusion rendering, which makes the region of the object in the AR view depth-aware transparent.

**Figure 8:**
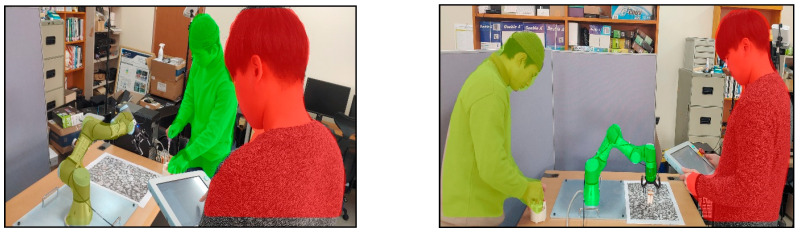
3D spatial relation among segmented instances.

As 3D spatial relation can be derived from the 3D meshes of segmented instances, the location and orientation of a visual information panel or object can be easily synchronized with the detected real facility. Figure 8 shows the segmented objects and their spatial relations according to the different viewpoints of the worker. The segmented object with the red color is located near the worker, and that with the green color is located far from the worker.

**Figure 9:**
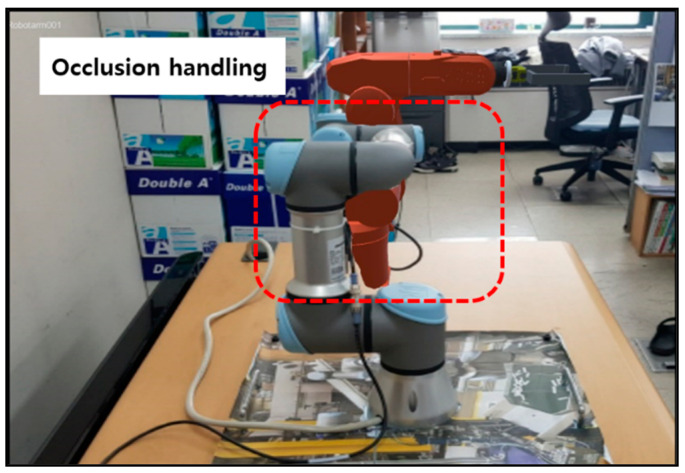
Handling of incorrect occlusion in Figure 1b using 3D spatial relation.

**Figure 10:**
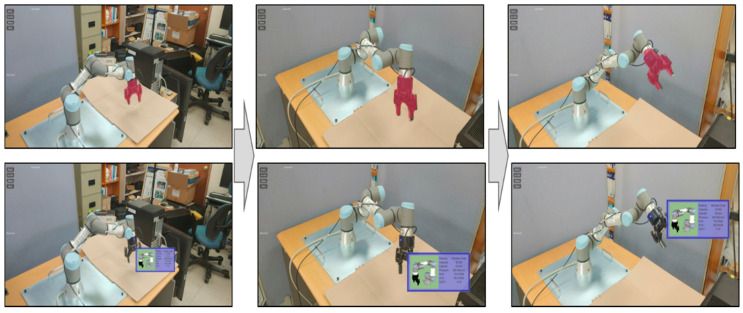
Adaptive visualization for a dynamically moving robot in industrial AR; the gripper of UR3 can also be detected, segmented, and annotated consistently.

Figure 9 and Figure 10 show how the proposed AR can handle the occlusion properly and can provide depth-aware and intuitive visualization to the worker. The manufacturing information corresponding to the actual facility can be visualized dynamically and adaptively around the facility according to its location and spatial relation. As shown in Figure 9, the virtual object (robot) on the rear side is occluded adequately by the UR3 robot closer to the worker.

In particular, even if the actual equipment is dynamically operating or moving by changing its configuration, the manufacturing information can be positioned around the equipment properly by updating its location, as the proposed approach can detect and segment facilities and predict their 3D depths and spatial information. On the other hand, when the existing AR marker is used, there is no problem in visualizing fixed and stationary equipment, but visual mismatching occurs when the position and configuration of the equipment are changed. Figure 10 shows how to effectively perform the manufacturing information visualization for the gripper attached to the UR3 robot using the proposed AR method. As the UR3 robot moves to grip a product or a component, the configuration of the robot changes the position and orientation of the gripper. Nevertheless, the proposed approach can recognize the movement of the gripper and consistently augments the relevant information, which can solve the common visual mismatch in the AR environment. These examples prove the effectiveness and advantage of the proposed approach.

## 4. System Implementation and Comparative Evaluation

We implemented the proposed industrial AR, which can segment actual facilities and predict their 3D depths from the AR view for task assistance. Instance segmentation and depth prediction can run on either TensorFlow [43] or Pytorch [44] in Windows 10. Two GPUs with GTX 1080 Ti architecture were used for training and testing. One GPU is assigned to instance segmentation, and the other to depth prediction. The industrial AR application was developed using Unity3D [45]. For the evaluation of the industrial AR, an additional seven classes based on pre-trained weights of the COCO dataset [46] were trained and tested, including UR3 robot, gripper, press machine, etc. Each class was trained with 250 segmented images. The learning rate was 0.001, and the epoch was 100. For training depth prediction, 130,000 images were used [38,39]. The learning rate was 0.0002, and the epoch was set to 30. To verify the feasibility and viability of the proposed approach, it was implemented in two environments, a controlled laboratory environment and a testbed for a smart factory.

We compared the proposed industrial AR with conventional AR marker-based methods (e.g., planar and cylindrical markers) using a popular Android-based smartphone (Samsung Galaxy S9^TM^) and another AR method using a smartphone with an RGB-D camera (Google Tango^TM^). In the case of AR marker-based methods, the 3D model is overlaid on the AR marker when the AR method recognizes the marker. In the case of the method using the RGB-D camera, the outline of the facility is segmented using the Mask R-CNN used in the proposed approach, but only the point cloud data corresponding to the segmentation are extracted from among those obtained by the RGB-D camera. Two experiments were conducted to evaluate three methods. First, we examined the effectiveness of the recognition of real equipment according to the variable distance. Different color gradation is used to represent the distance from the AR system. For example, the color becomes darker when the detected object is far from the user. Second, we evaluated the recognition of dynamically moving equipment. Note that one of the main advantages of the proposed industrial AR visualization is that it does not require a depth sensor.

### 4.1. Recognizable Distances of Real Objects

**Figure 11:**
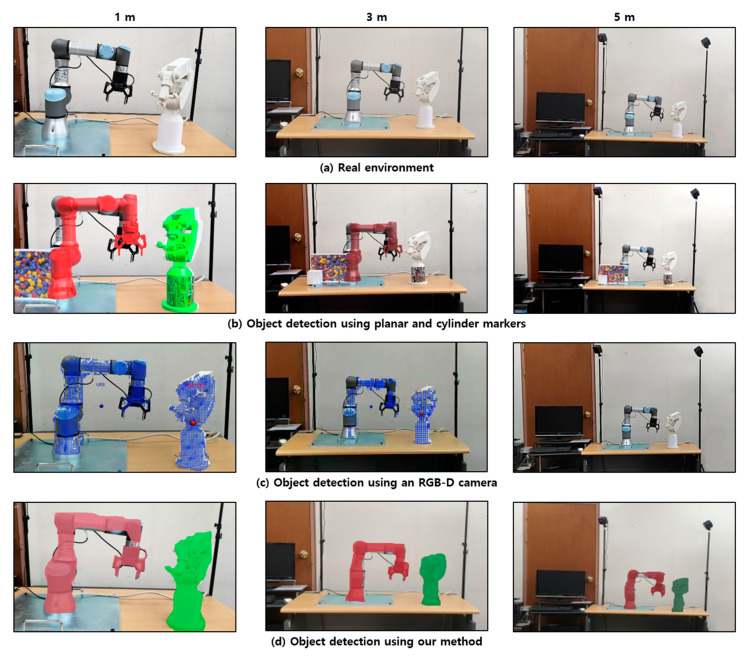
Comparative evaluation of the proposed approach.

We evaluated whether each method can detect and superimpose a virtual model onto corresponding real equipment consistently and effectively depending on the distance between the AR camera and the equipment. For this purpose, each AR system was located at a distance of 1, 3, or 5 m from the UR3 robot and another robot prototype created using a 3D printer, as shown in Figure 11.

First, the cylindrical AR marker-based method showed the worst performance. On the other hand, the proposed method showed the best performance without AR markers and an RGB-D sensor. When a cylindrical AR marker was attached to the physical object, the marker was recognized at a distance of 1 m, but the marker was not identified at distances of 3 and 5 m (Figure 11b). The size of the cylinder-shaped AR marker was 8.5 cm in diameter and 7 cm in height due to the geometric shape of the prototype. The rectangular AR marker was recognized at distances of 1 m and 3 m, but the AR marker was not recognized at a distance of 5 m. Note that the marker was attached to the perpendicular direction to the AR camera for easy recognition.

**Figure 12:**
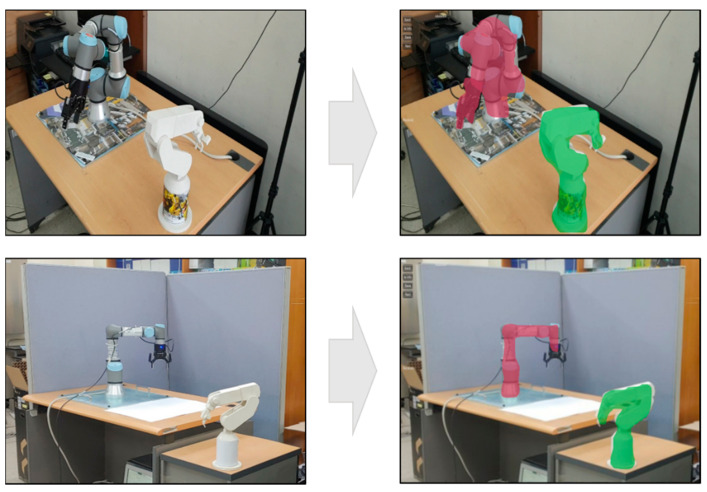
Recognition of physical objects with different distances (depth perception).

However, this attachment was not normal. When the marker was attached to the floor, its recognition rate became worse. As shown in Figure 11c, when the real object was detected using the same instance segmentation using the RGB-D camera, depth information of the actual equipment was only acquired at distances of 1 m and 3 m. In the case of the RGB-D camera [10], the range of obtaining the depth information is about 50 cm to 4.5 m. Furthermore, the accuracy of the depth perception depends on the material type and distance too much. Unlike the conventional AR marker-based and RGB-D sensor-based approaches, the method proposed in this study recognized real facilities more consistently and accurately at all distances, as shown in Figure 11d. In addition, it can also construct the 3D spatial relations among the detected facilities. Figure 12 shows that the proposed approach can effectively find the spatial relations, although the interesting objects are separated a lot concerning the spatial relation in Figure 11.

Table 1 shows quantitative results for measuring the recognition rate and processing time of the experiment in Figure 11. For 5 m, the marker-based method cannot recognize the objects at all, although it can run in real-time. In particular, the method cannot detect the cylindrical marker, even at 3 m. Furthermore, the existing marker-based methods cannot deal with occlusion and visual mismatch properly. The RGB-D camera cannot also recognize objects at a distance of 5 m. Only the proposed approach can recognize objects correctly. Note that most mobile devices such as smartphones and tablets on the market today do not have RGB-D cameras so that the proposed approach could be more practical. Due to the processing of deep learning and mesh generation, it takes about one second for AR visualization. Nevertheless, the proposed approach can deal with incorrect occlusion and visual mismatch that prohibit the worker from understanding the manufacturing situation correctly and effectively. Concerning the advantages of each approach, the proposed method and the existing AR method can be used at the same time so that they can be switched to each other as needed, thereby complementing each other.

### 4.2. Handling Dynamically Moving Objects

**Figure 13:**
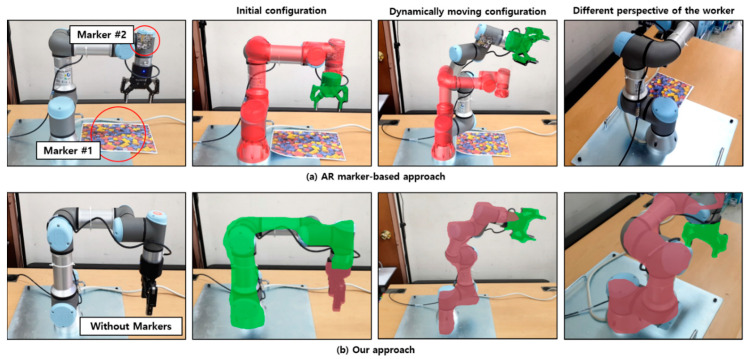
Handling a dynamically moving robot in AR.

We have also evaluated the capability for handling dynamically moving objects. As shown in Figure 13, two AR markers were used in the marker-based method. The first marker was attached to the body of the UR3 robot, and the other was attached to the gripper to detect them separately during the movement. On the other hand, the proposed approach does not need any AR makers.

Note that one of the advantages of using the AR marker is to detect actual facility fast and accurately. However, when the configuration of the facility is changed, or the facility is moving, it is almost impossible to detect parts or components attached to the facility without receiving additional information through other sensors. Furthermore, it is still difficult to detect and segment the facility itself, and their components as AR markers might be occluded when the facility and their parts are moving, as shown in Figure 13. Such problems make it difficult to attach AR markers on the dynamically moving equipment, which may prevent the industrial worker from performing the task properly. On the other hand, the proposed approach can detect and segment the dynamically moving equipment with only single AR images. Besides, the equipment can be recognized at various perspectives even if the worker’s position changes. These assessments prove the novelty and advantage of the proposed approach.

## 5. Industrial Applications

To verify the advantages and applicability of the proposed industrial AR, several proof-of-concept applications were implemented.

### 5.1. Manufacturing Information Visualization

**Figure 14:**
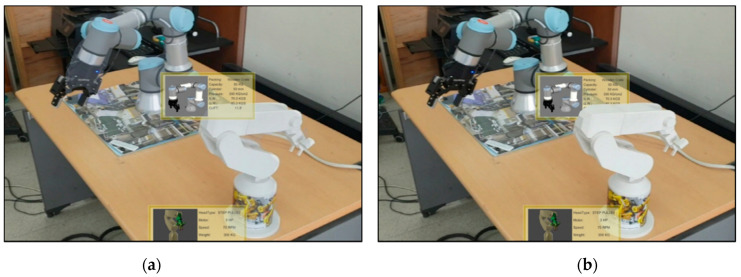
Handling incorrect occlusion during the robot’s operation with the proposed approach: AR information can be displayed in a depth perception way: (**a**) a conventional AR visualization; (**b**) the proposed AR visualization

The manufacturing information visualization using the proposed industrial AR was implemented using robots in a controlled laboratory environment. In particular, the UR3 robot can move to pick up a part and place it to a specified location. During the robotic operation, the kinematic configuration of the robot is changed. However, the conventional AR cannot overcome the inherent occlusion problem shown in Figure 14a with the AR marker, as it cannot determine the spatial relations between physical objects. Furthermore, it cannot seamlessly provide consistent visualization when the kinematic configuration is changed, even with multiple AR markers. The main reason is that the existing AR technology superimposes virtual information on the AR image. On the other hand, as shown in Figure 14b, the proposed approach can handle occlusion properly so that it can provide consistent views to the worker.

**Figure 15:**
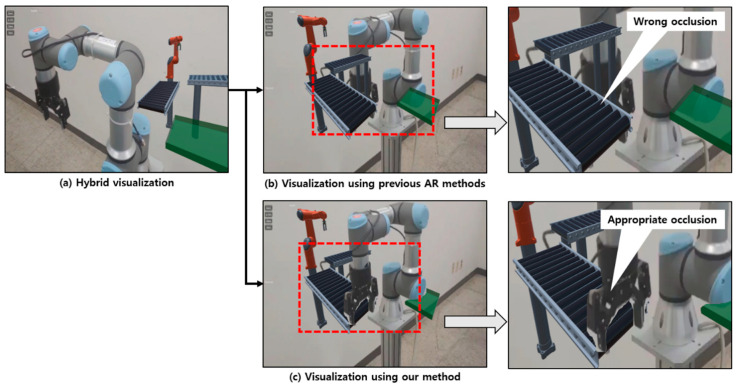
Dynamic modeling of a manufacturing layout using the proposed industrial AR.

The proposed AR method can also be applied to a hybrid AR environment that fuses virtual and real objects. For example, it can be effectively applied to a process layout design where a new facility will be added to examine possible layouts or simulations with existing facilities. As can be seen in Figure 15, if a new robot or facility is expected to be installed in the current layout, connected to the current UR3, a virtual robot or facility can be augmented next to the UR3. However, in the existing AR technology, there is no visual problem in the case where the virtual robot is separated from UR3 (Figure 15a), but when the viewpoint of the worker is changed while inspecting the new layout, the virtual facility is augmented in front of UR3, which causes an occlusion problem, as shown in Figure 15b. As the previous AR cannot estimate the depth perception with AR images, visual mismatch and occlusion issues may occur between the virtual model and the actual facility, which may make it difficult to conduct a proper preliminary review. On the other hand, as shown in Figure 15c, the proposed AR method can solve those problems by applying instance segmentation and depth prediction, even if virtual and real objects coexist.

**Figure 16:**
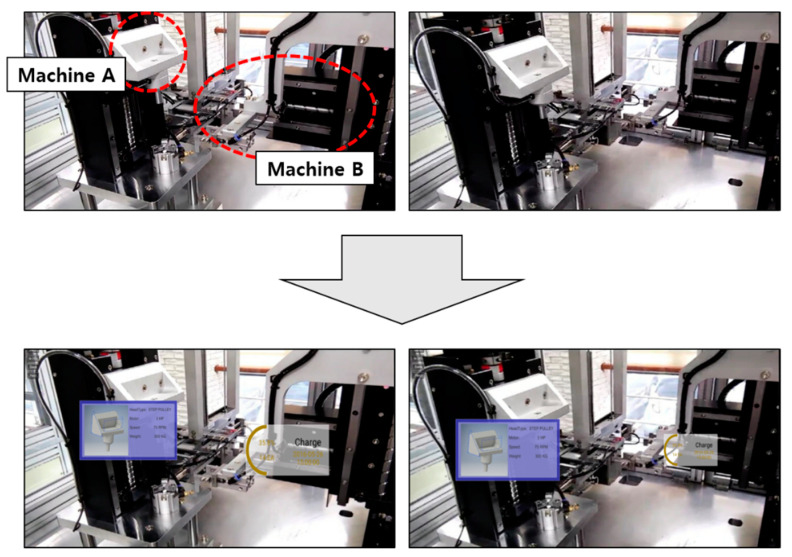
Application of the proposed industrial AR to an assembly line in a smart factory.

The second application was applied to the testbed of a smart factory (Figure 16). The testbed used to this study was built in a Gumi city of Korea and produced various brushless direct current (BLDC) motors in a flexible assembly line [28]. This implementation was applied to the visualization of the manufacturing information in the module for sub-assembling bearings of BLDC motors. With the help of the proposed industrial AR method using the smart device, the worker can easily recognize assembling machines and tools such as press machine and gripper, as shown in Figure 16. As the outlines of the machine and tool can be segmented, and their 3D spatial relationship can be estimated through depth prediction, real-time monitoring data and related information can be retrieved from manufacturing database, and they can be seamlessly augmented onto physical facilities without occlusion problem and visual mismatch. For example, manufacturing information retrieved from DB, such as production schedule, production quantity by item, and related charts, is superimposed adaptively around the machine and gripper. Additionally, as shown in Figure 16, although the machine configuration is changed during the assembly, the proposed approach supports consistent visualization, and thus, corresponding manufacturing information can be visualized dynamically in the correct position. Therefore, the worker can receive more accurate information.

### 5.2. Different Applications Using the Pre-Trained Network

**Figure 17:**
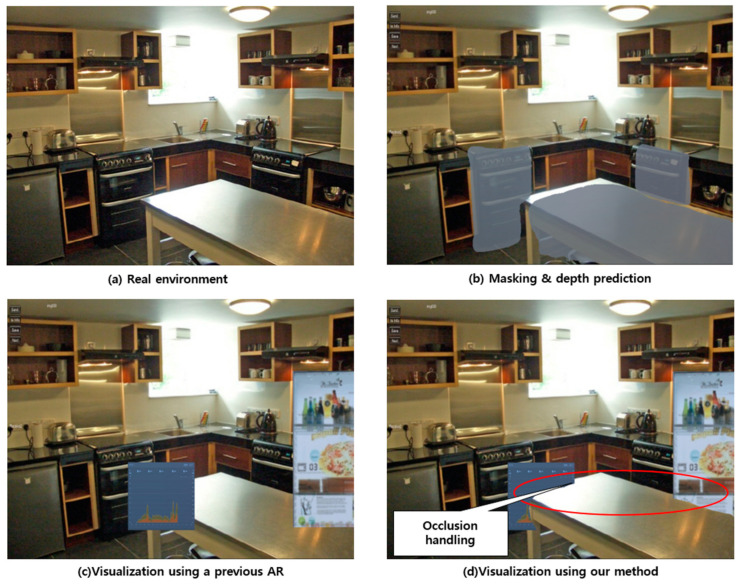
Application of the proposed approach to IoT visualization in a smart home.

In addition to applying the proposed industrial AR to manufacturing information visualization, we have found that it can be applied to the visualization of smart appliances or devices in a smart home. In this application, a pre-trained network was utilized. As shown in Figure 17a, information on home appliances connected to Internet of Things (IoT) at home can also be visualized in the augmented reality environment. Figure 17c shows a typical result of applying existing AR technology. Information on the oven can be augmented, but the table is occluded by the AR visualization. However, Figure 17b shows the visualization of the segmentation and depth information of the appliances obtained through the proposed method. Furthermore, Figure 17d shows the proposed visualization of an appliance information through handling occlusion properly using segmented 3D point cloud and predicted depth information. Unlike the existing AR technology, we can see the information of the appliance occluded by the table.

**Figure 18:**
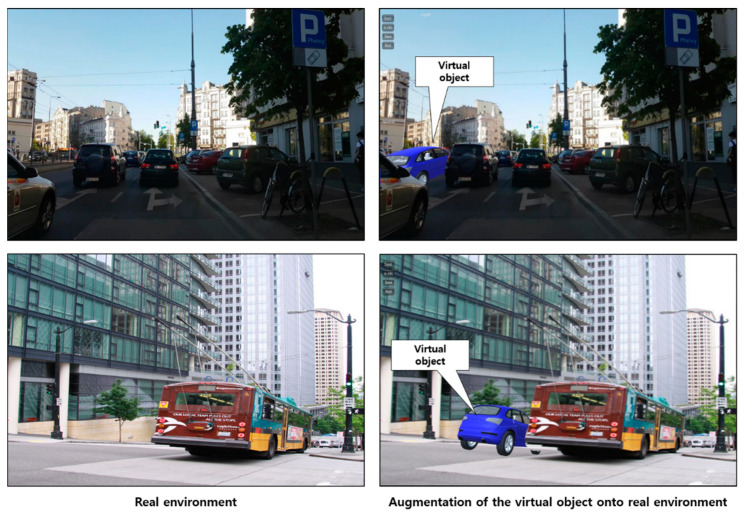
Application of the proposed approach to street visualization.

The second application is to visualize augmented virtual objects between real objects in RGB images. As shown in Figure 18, the proposed method can support seamless visualization of a virtual car between real cars using instance segmentation and depth prediction in the street. The virtual car is partially occluded by a real car or bus closer to the camera view, and the car far away from the camera view is also properly occluded by a virtual car.

## 6. Discussion

Existing AR methods have difficulties in handling incorrect occlusions and visual mismatches and in estimating depth from AR images, which prevents the industrial worker from conducting manufacturing tasks effectively. To solve these inherent problems in existing AR methods, this study proposed a new hybrid approach to industrial AR for complementing existing AR methods using deep learning-based facility segmentation and depth prediction without AR markers and a depth camera.

Several case studies and experimental analysis in Section 5 verified the advantage of the proposed approach compared with existing AR methods. In particular, handling incorrect occlusions and visual mismatches can make the worker understand the manufacturing situation more effectively and intuitively. Note that the proposed approach does not require the RGB-D camera.

**Figure 19:**
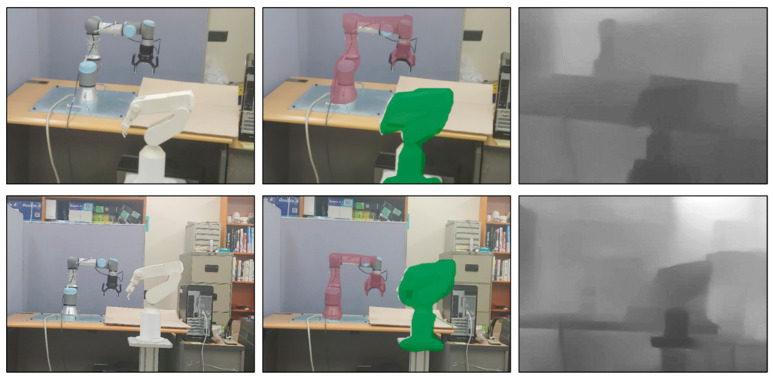
Limitation in depth prediction.

Nevertheless, there is some room for improvement and investigation. The depth prediction does not always construct 3D depth maps accurately depending on the background color and its complexity, as shown in Figure 19 (right). Nevertheless, by combining instance segmentation and depth prediction, it might be possible to estimate 3D spatial relations between detected objects correctly, since instance segmentation and depth prediction can complement each other. However, it is necessary to improve the capability of the depth prediction by training a variety of data and improving the deep learning network. In addition, since the mobile device has hardware and software limitations, the deep learning methods run remotely. Although it is expected that deep learning can run on a mobile device, it is still necessary to devise a more efficient deep learning architecture such as an end-to-end deep learning model that supports both instance segmentation and depth prediction to improve the speed and accuracy.

Nevertheless, the proposed approach and an existing AR method complement each other. That is, for ordinary situations, the existing AR method is used, since it can support real-time marker tracking. However, as the existing AR cannot handle incorrect occlusions and visual mismatches, the worker can switch to the proposed approach to understand the situation more effectively and intuitively.

## 7. Conclusions

This paper proposed a new industrial AR method that can handle visual mismatches and incorrect occlusions of previous AR methods in representing manufacturing information by applying deep learning-based facility segmentation and depth prediction. From a single AR image, object instances are detected and segmented. At the same time, depth prediction is applied to the same AR image. Then, a segmented 3D point cloud for each detected object can be easily generated. Besides, a 3D triangular mesh is constructed from the segmented 3D point cloud, which makes it possible to find 3D spatial relations among the detected objects and render them in the 3D AR space rather than the image-based AR space. The constructed 3D mesh and spatial relation are used to solve visual mismatch and occlusion problems. Furthermore, the proposed approach can provide consistent AR visualization regardless of the movement of the physical object. Therefore, manufacturing information can be provided to the worker more intuitively and situation-dependently based on the estimated 3D spatial relation and depth perception. Note that the proposed approach can be utilized as a hybrid or complementing function to existing AR methods, since it can be activated whenever the industrial worker requires handing of visual mismatches or occlusions. For another possible storyboard, a facility manager can utilize the proposed approach, since the manager has difficulty in monitoring and maintaining the facility by looking at its outside or surface. In such a case, it would be more helpful to combine various IoT sensor data with the proposed AR system.

In future research, we will improve the deep learning network architecture because it is affected by the performance of the deep learning network. In addition, the proposed approach will be applied to various industrial environments. Furthermore, we will also design more storyboards of actual and realistic industrial situations.

## Figures and Tables

**Table 1:** Quantitative results for measuring recognition rate and processing time (the number of tests: twenty).

Methods	Distance	Recognition Rate (20 Times) [Successful Trial/Total Trial (Recognition Rate)]	Average Process Time(s)
Planar and cylindrical markers	3 m	Planar marker: 20/20(100%)	Real-time
		Cylindrical markers: 0/20(0%)	-
	5 m	0/20(0%)	-
RGB-D camera	3 m	20/20(100%)	0.801
	5 m	0/20(0%)	-
Our Method	3 m	20/20(100%)	1.191
	5 m	18/20(90%)	0.994

## Data Availability

Not Applicable.
